# Identification of mitochondrial function and programmed cell death associated key biomarkers and the circRNA-miRNA-mRNA regulatory network in systemic lupus erythematosus

**DOI:** 10.3389/fmolb.2025.1586294

**Published:** 2025-04-14

**Authors:** Junjie Cao, Aifang Li, Hui Zhou, Yujie Yan, Gaiying Luo

**Affiliations:** ^1^ Department of Laboratory Medicine, Xi’an Fifth Hospital, Xi’an, Shaanxi, China; ^2^ Department of Laboratory Medicine, Xi’an Chest Hospital, Xi’an, Shaanxi, China; ^3^ Department of Cardiovascular, Xi’an Fifth Hospital, Xi’an, Shaanxi, China; ^4^ Medical Collage, Xi’an Peihua University, Xi’an, Shaanxi, China

**Keywords:** mitochondrial function, programmed cell death, systemic lupus erythematosus, bioinformatics, machine learning

## Abstract

**Objectives:**

Systemic Lupus Erythematosus (SLE) is a highly heterogeneous autoimmune disease with complex pathogenic mechanisms. Mitochondrial function and programmed cell death (PCD) play important roles in SLE. This study aims to screen biomarkers related to mitochondrial function and programmed cell death in SLE and analyze their underlying mechanisms.

**Methods:**

SLE-related databases were derived from the GEO database, where three SLE databases were merged into one database as the training set. Genes related to mitochondrial function and PCD were sourced from the MitoCarta 3.0 database. Key genes were identified through bioinformatics and machine learning, and their expression levels and diagnostic efficacy were validated using two SLE-related datasets as the validation set. The relationship between diagnostic genes and immune cells was analyzed through CIBERSORT immune infiltration analysis. Diagnostic genes-related miRNAs were predicted using online databases. Differential circRNAs were screened in SLE circRNA datasets, and the relationship between circRNAs and miRNAs is predicted through circbank, finally constructing a circRNA-miRNA-mRNA ceRNA regulatory network.

**Results:**

From the 448 differential genes in the SLE training set, two key genes, IFI27 and LAMP3, were identified through machine learning and WGCNA. Enrichment analysis revealed that they were mainly enriched in pathways such as cell cycle, systemic lupus erythematosus, cytosolic DNA sensing pathway, toll-like receptor (TLR) signaling pathway and nod-like receptor (NLR) signaling pathway. Immune infiltration analysis found that compared with normal group, 11 immune cells were differentially expressed, with IFI27 related 9 types of immune cells and LAMP3 related 10 types of immune cells. The final constructed circRNA-miRNA-mRNA ceRNA regulatory network consists of 2 mRNAs, 5 miRNAs, and 4 circRNAs.

**Conclusion:**

Our study ultimately identified two biomarkers (IFI27 and LAMP3) related to mitochondrial function and programmed cell death that play an important role in SLE. In the future, IFI27 and LAMP3 have the potential to become important biomarkers in the diagnosis and treatment of SLE. Their role in the immune response may provide new strategies for the treatment of SLE.

## 1 Introduction

Systemic Lupus Erythematosus (SLE) is a systemic autoimmune disease characterized by dysregulation of innate and adaptive immunity, abnormal production of autoantibodies, and the formation and deposition of immune complexes in various organs and tissue (Siegel and Sammaritano, 2024). The production of anti-double-stranded DNA (anti-ds-DNA) is a notable feature of SLE (Anis et al., 2023). Excessive cell death and failure in the clearance of dead cells were considered one of the main pathogenic mechanisms leading to the generation of autoantigens and the initiation of autoimmune responses. Numerous studies have indicated that different forms of Programmed Cell Death (PCD) play a significant role in the pathogenesis of SLE (Xu Y. et al., 2022). The dysregulation of PCD pathways and defects in the clearance of dead cells can promote the release of Damage-Associated Molecular Patterns (DAMPs) in SLE, amplifying inflammation and immune responses, generating autoantigens, and causing tissue damage (Alvarez and Vasquez, 2017).

PCD is an essential physiological process that plays a critical role in maintaining tissue homeostasis and eliminating damaged or unwanted cells. PCD can occur through various mechanisms, including: Apoptosis, Anoikis, Autophagy, Alkaliptosis, Cuproptosis, Entosis, Entotic cell death, Immunogenic cell death, Ferroptosis, Lysosome-dependent cell death, Methuosis, Necroptosis, Netotic cell death, NETosis, Oxeiptosis, Pyroptosis, Parthanatos, Paraptosis (Yan et al., 2024).

Mitochondria are organelles with a double membrane and play a crucial role in energy production, iron homeostasis, and the biosynthesis of lipids, amino acids, and nucleic acids. Additionally, mitochondria are key in regulating cellular signaling pathways and controlling PCD (Nguyen et al., 2023). Moreover, mitochondria possess many DAMPs that can initiate various inflammatory signaling pathways (Marchi et al., 2023). Disruption of mitochondrial morphology, such as changes in shape, size, or cristae organization, can impair normal mitochondrial function and trigger PCD (Gibellini and Moro, 2021). Structural abnormalities may affect the release of pro-apoptotic factors within the mitochondria, leading to caspase activation and subsequent apoptosis (Vringer and Tait, 2023). Mitochondrial function is also closely related to the mechanisms of PCD in SLE. In SLE, mitochondrial DNA (mtDNA) from neutrophils is highly susceptible to oxidation by mitochondrial reactive oxygen species and can be released from the mitochondria. Furthermore, mtDNA can also be released into the extracellular environment. Extracellular mtDNA activates plasmacytoid dendritic cells and CD4^+^ T cells, which is critical for the pathogenesis of SLE (Mobarrez et al., 2019). Oxidative and nitrosative stress resulting from mitochondrial dysfunction may serve as pathobiological signals for increased apoptosis/necrosis, the formation of multiple new antigens, and immune dysregulation in SLE patients (Miao et al., 2023).

Circular RNA molecules (circRNAs) are a class of non-coding RNA molecules that exist *in vivo* without a 5′terminal cap and a 3′terminal poly(A) tail, and form a covalently closed circular structure. circRNAs can bind endogenously with miRNAs to regulate gene expression. The circRNA-miRNA-mRNA network constitutes the mechanism of the competitive endogenous RNA (ceRNA) network. It has been found that various abnormally expressed circRNAs play a potential role in SLE (He et al., 2024). In addition, the infiltration of immune cells determines the microenvironment of the disease, thereby affecting the immune response, which is key to the pathogenesis and treatment of immune-related diseases. The dysregulation of various immune cells, including B cells, CD4^+^ T cells, follicular helper T cells, and dendritic cells, is related to the pathogenesis of SLE (Xu L. et al., 2022).

Current research on the pathogenesis and early diagnosis of SLE is still insufficient. This study, based on bioinformatics and machine learning, starts from the perspectives of mitochondrial function and PCD, to screen and study genes related to mitochondrial function and PCD in SLE from the Gene Expression Omnibus (GEO) database. By using Weighted Gene Co-Expression Network Analysis (WGCNA) and machine learning to select key genes, and by verifying the genes and examining clinical samples to explore their potential as biomarkers. We further analyzed the immune infiltration status and the correlation with key genes using CIBERSORT (Cell-type Identification By Estimating Relative Subsets Of RNA Transcripts) to explore the relationship between immune cells and key genes. Finally, we predicted related miRNAs through the miRNet database and predicted related circular RNAs through the circbank database, and analyzed the GEO database of SLE’s circular RNAs to construct a circRNA-miRNA-mRNA network, discussing the possible regulatory pathways of key genes. This study analyzed three gene expression datasets (GSE4588, GSE50772, and GSE81622) downloaded from the GEO database, identified two important hub genes, and clinically validated the key genes. It lays the foundation for discovering new indicators for diagnosing and monitoring SLE, and provides new ideas and targets for the prevention and treatment of SLE.

## 2 Materials and methods

### 2.1 Study design

**FIGURE 1:**
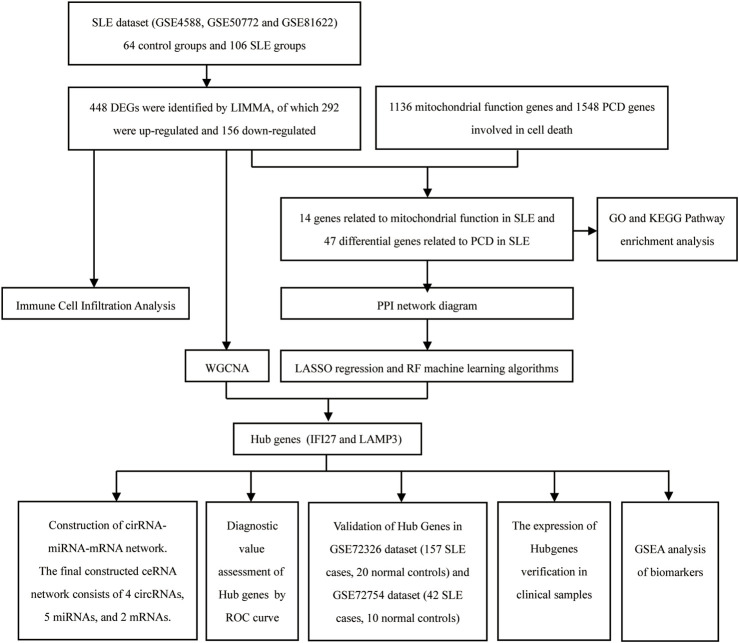
The flowchart of this study.

Using *Homo sapiens* as the object of study, 6 data sets (GSE4588, GSE50772, GSE81622, GSE72326, GSE72754, and GSE84655) including gene expression data for SLE and normal peripheral blood mononuclear cells, were downloaded from the Gene Expression Omnibus database (https://www.ncbi.nlm.nih.gov/geo/). Three SLE related datasets (GSE4588, GSE50772 and GSE81622) were integrated as SLE dataset. The SLE dataset contained samples from 64 control groups and 106 SLE groups. The GSE72326 and GSE72754 datasets served as validation sets, and the GSE84655 dataset was used to screen for differentially expressed circular circRNAs in SLE. A total of 1,136 mitochondrial function genes and 1548 PCD genes involved in 18 cell death were derived from the MitoCarta 3.0 database (Yan et al., 2024; Li et al., 2023). LIMMA was utilized to screen for differentially expressed genes (DEGs) between SLE and the normal group, then these DEGs were intersected with genes related to mitochondrial function and PCD to identify shared DEGs. Sangerbox platform was applyed to perform Gene Ontology (GO) and Kyoto Encyclopedia of Genes and Genomes (KEGG) enrichment analysis, as well as Protein-Protein Interaction (PPI) network analysis on the shared DEGs. Lasso regression and random forest was employed for hub gene screening, and WGCNA was used to select co-expression module genes in SLE, taking the intersection of genes from the three methods as the hub genes. Further, Receiver Operating Characteristic (ROC) curve was employed to test the diagnostic value of the hub genes for SLE. The CIBERSORT algorithm was employed to analyze and compare the distribution differences of immune cells between SLE and the normal group. The hub genes were validated in the validation datasets GSE72326 and GSE72754. In the GSE84655 dataset, differentially expressed circRNAs (DECs) in SLE were selected, and analyzed through online databases and SLE’s cirRNA microarray chips. We predicted the relevant miRNAs and cirRNAs. Using Cytoscape software, a regulatory network of cirRNA-miRNA-mRNA was constructed to explore the potential interactions and regulatory mechanisms in SLE. The flowchart of this study was shown in Figure 1.

### 2.2 Differentially expressed gene screening

First, all three raw SLE datasets were background-calibrated, normalized, and log2-transformed using the “affy” package in the R software program. Limma package was used to identifying DEGs and DECs. The setting criteria was as follows: |log2 Fold change (FC)| ≥0.585 for SLE dataset, |log2 Fold change (FC)|≥1 for circRNA dataset and p-value <0.05. Volcanic maps were used to show DEGs, and heat map was used to show the top 50 genes with the most significant expression differences.

### 2.3 Functional enrichment analysis and Gene Set Enrichment Analysis

Functional enrichment analysis was conducted via the Sangerbox platform (http://vip.sangerbox.com/). p < 0.05 was set as the criteria. In this study, GO and KEGG analyses were performed respectively based on the intersection of DEGs for SLE and genes of Mitochondrial function, and the intersection of DEGs for SLE and the genes of PCD.

Gene Set Enrichment Analysis (GSEA) was used to assess the distribution trend of genes from a pre-defined gene set in the gene list ranked with phenotypic relevance, so as to judge their contribution to phenotype. We got the GSEA software (version 3.0) from the GSEA web site (http://software.broadinstitute.org/gsea/index.jsp), and download the c2. Cp. Kegg. V7.4. symbols. gmt subset from Molecular Signatures Database (http://www.gsea-msigdb.org/gsea/downloads.jsp) to evaluate the relevant pathways and molecular mechanisms. Based on the gene expression profile and phenotype grouping, the minimum gene set was 5, the maximum gene set was 5,000, 1,000 resampling runs were performed. P value of <0.05 (as needed) and a FDR of <0.25 (as needed) were considered statistically significant.

### 2.4 Protein–protein interaction network construction

Protein-protein interaction (PPI) network was established by the String database (version 11.5; www.string-db.org). The STRING database is a powerful online bioinformatics tool for retrieving and analyzing PPI. The STRING database provides a composite score based on evidence from multiple sources, including experimentally determined interactions, bioinformatics predictions, literature mining, and more. The overall score reflects the degree of confidence in the interaction between the two proteins. In STRING, a composite score of more than 0.4 is generally considered statistically significant, meaning that some form of interaction is likely to exist between the two proteins. Cytoscape software was adopt to construct this PPI network.

### 2.5 Weighted gene Co-Expression network analysis and module gene selection

The Weighted Gene Co-expression Network Analysis (WGCNA) is a powerful tool for constructing gene co-expression networks and identifying functional modules. First, calculate the median absolute deviation (MAD) for each gene, which is a robust measure of variability. Genes with MAD values in the bottom 50% were considered to have low variability and were often removed from the analysis. Second, remove unwanted genes and samples using the “good Samples Genes” function to construct a scale-free co-expression network. Third, determine the Soft Threshold Power (β) to calculate the adjacency matrix, which represents the degree of correlation between gene expression profiles. And transform adjacency to Topological Overlap Matrix (TOM) to assess the topological overlap between gene pairs. This matrix was used to determine gene significance and module membership. Forth, perform Hierarchical Clustering to group genes into modules based on their topological overlap. The dynamic tree cutting method was then applied to identify distinct modules within the network. Finally, assess the correlation between gene significance and module membership to identify gen good Samples Genes es that were highly representative of their respective modules. Once the modules were identified, extract the genes within each module for further functional analysis and exploration of their biological significance. To identify the most relevant gene module in SLE, WGCNA was adopted. Based on scale independence and mean connectivity, we selected β = 8.087 as the soft threshold.

### 2.6 Machine learning

The Least Absolute Shrinkage and Selection Operator (LASSO) is an estimation method that minimizes the sum of squared residuals under the constraint that the sum of the absolute values of the regression coefficients is less than a constant, which can produce some regression coefficients that were exactly zero, leading to an interpretable generalized linear model. In this study, LASSO regression was used to select candidate genes with diagnostic significance for SLE mitochondrial function and PCD, using the “glmnet” package for LASSO regression analysis of these candidate genes. Random Forest (RF) is a supervised, ensemble learning algorithm based on decision trees, and in this study, the “randomForest” package in R was used to implement RF, with genes selected based on an importance score greater than 2.0. The intersection genes of LASSO, Random Forest and WGCNA were considered as candidate hub genes.

### 2.7 Receiver operating characteristic evaluation

Use the pROC package in R software to plot the ROC curve and calculate the area under curve (AUC) and 95% confidence interval (CI) to quantify its value. ROC curve was used to evaluate the diagnosis of candidate genes for mitochondrial function and PCD in SLE, and AUC>0.7 was considered to be the ideal diagnostic value.

### 2.8 Validation of hub genes expression in other data sets

The mRNA expression of identified hub genes was verified in GSE72326 and GSE72754. The GSE72326 dataset contains 157 SLE and 20 normal. GSE72754 consists of 42 SLE and 10 normal. The comparison between the two sets of data was performed with the T-test. P-value <0.05 was considered significant.

### 2.9 Immune infiltration analysis

CIBERSORT is a computational method that uses tissue gene expression profiles to identify the proportions of various immune cells. In this study, it was conducted to determine the immune cell proportions between SLE and normal group. Immune cell infiltration analysis was conducted using the “Cibersort” R package. Violin plots were used to illustrate the proportions of different types of immune cells in SLE and normal group. The “corrplot” R package was used to create correlation graphs for 22 types of infiltrating immune cells. Subsequently, Spearman’s correlation analysis was performed between biomarkers and differential immune cells.

### 2.10 CircRNA-miRNA-mRNA network in SLE

Human microRNA Disease Database (HMDD, www.cuilab.cn/hmdd) is a database that includes experiment-supported evidence for human miRNA and disease associations. HMDD v4 was used in our work to search for the SLE-related microRNAs (miRNAs). miRNet (www.mirnet.ca) is an easily accessible web-based tool that offers statistical, visualization, and network-based approaches to demonstrate the functionality and regulatory mechanisms of miRNAs. It was used to predict miRNA from mRNAs in our study. Venn diagram was used to overlap the SLE-related miRNAs and miRNAs predicted from mRNAs in our study. The circRNA regulating miRNA was predicted by circbank database (www.circbank.cn) and intersected with circRNA related to SLE. The visualization of cirRNA-miRNA-mRNA regulatory network was constructed using Cytoscape software.

### 2.11 Expression verification of key DEGs by real-time quantitative PCR

Fifteen patients with SLE who met the 1997 American College of Rheumatology (ACR) criteria and ten age and sex matched health volunteers were selected from Xi ‘an Fifth Hospital. All samples were approved by Ethics Committee of Xi ‘an Fifth Hospital. Peripheral Blood Mononuclear Cell (PBMC) was isolated by Ficoll density gradient centrifugation method, and total RNA was extracted by TRIzol (Ambion, Austin United States). The extracted RNA was reverse-transcribed into cDNA using the PrimeScript™ RT reagent Kit (Takara, Japan). mRNA expression levels of the key diagnostic genes IFI27 and LAMP3 in PBMC were detected by TB Green Premix Ex TaqTM II (Takara, Japan) RT‒PCR, and the experimental data obtained in the experiment were analyzed by 2^-△△CT^ method. The primer information was shown in Supplementary Table S1.

### 2.12 Statistical analysis

The ROC curve and t-test were performed by SPSS Version 23.0 (IBM Corporation, Armonk, NY, United States). p < 0.05 was considered as statistically significant.

## 3 Results

### 3.1 Data preprocessing

“genecard” was used for gene ID conversion of the gene expression profiles from the GSE4588, GSE50772, and GSE81622 datasets. The R software package “sva” was used to batch the above three data sets (see Supplementary Figure S1). A comprehensive GEO data set was created as a training set (a total of 170 samples, including 106 SLE cases and 64 healthy controls).

### 3.2 Identification of DEGs and functional enrichment analysis of SLE

**FIGURE 2:**
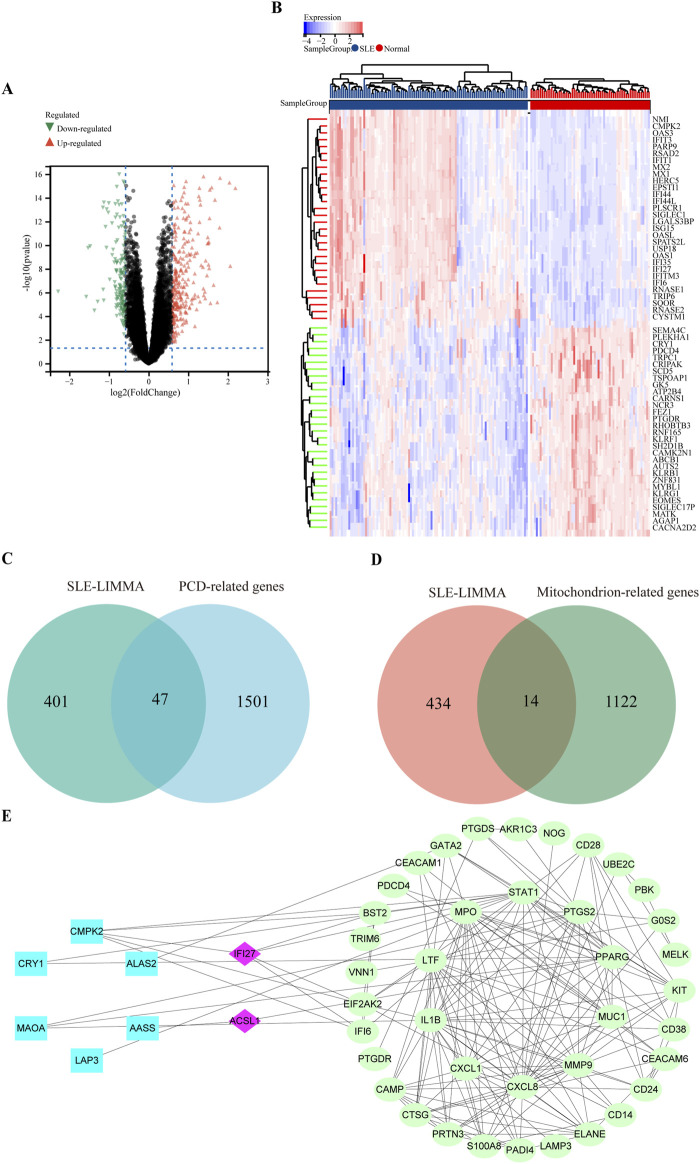
Volcano plot and heatmap for the DEGs identified from the integrated SLE dataset. **(A)** The volcano map of SLE dataset. Upregulated genes were marked in red; downregulated genes were marked in green. **(B)** The heat map of SLE dataset. Each row shows the DEGs, and each column refers to one of the samples of SLE cases or controls. The red and blue represent DEGs with upregulated and downregulated gene expression, respectively. **(C)** The overlap genes of mitochondrial function and SLE via Wenn diagram. **(D)** The overlap genes of PCD and SLE via Wenn diagram. **(E)** PPI network reveals that 46 genes interact with each other.

After standardizing the microarray results, 448 DEGs in the SLE dataset were identified, of which 292 were upregulated and 156 downregulated (Figure 2A). Figure 2B showed the clustering heat map of the top 50 DEGs. 448 SLE DEGs were intersected with 1,548 cell death related genes and 1,136 mitochondrial function related genes, respectively, and 47 differential genes related to PCD in SLE and 14 genes related to mitochondrial function in SLE were found (Figures 2C, D). A total of 58 differential genes (including three common genes) related to mitochondrial function and cell death were identified in SLE.

In order to analyze the biological functions and pathways involved in the common DEGs, GO and KEGG Pathway enrichment analysis were performed. For the 14 common DEGs of mitochondrial function in SLE, GO analysis results showed that these genes were mainly enriched in sulfur compound metabolic process, mitochondrion and oxidoreductase activity (Supplementary Figure S2B). In terms of KEGG Pathway, the three significant enrichment pathways were Metabolic pathways, Glycine, serine, and threonine metabolism and Arginine and proline metabolism (Supplementary Figure S2A). For the 47 common DEGs of PCD in SLE, GO analysis results showed that these genes were mainly enriched in PCD, apoptotic process and identical protein binding (Supplementary Figure S3B). In terms of KEGG Pathway, the two significant enrichment pathways were IL-17 signaling pathways and NF-kappa B signaling pathway (Supplementary Figure S3A).

Based on the STRING database, 46 of the 58 DEGs had interactions. A visual PPI network diagram was build using Cytoscape software. (Figure 2E).

### 3.3 Weighted gene Co-Expression network analysis and key module Identification

**FIGURE 3:**
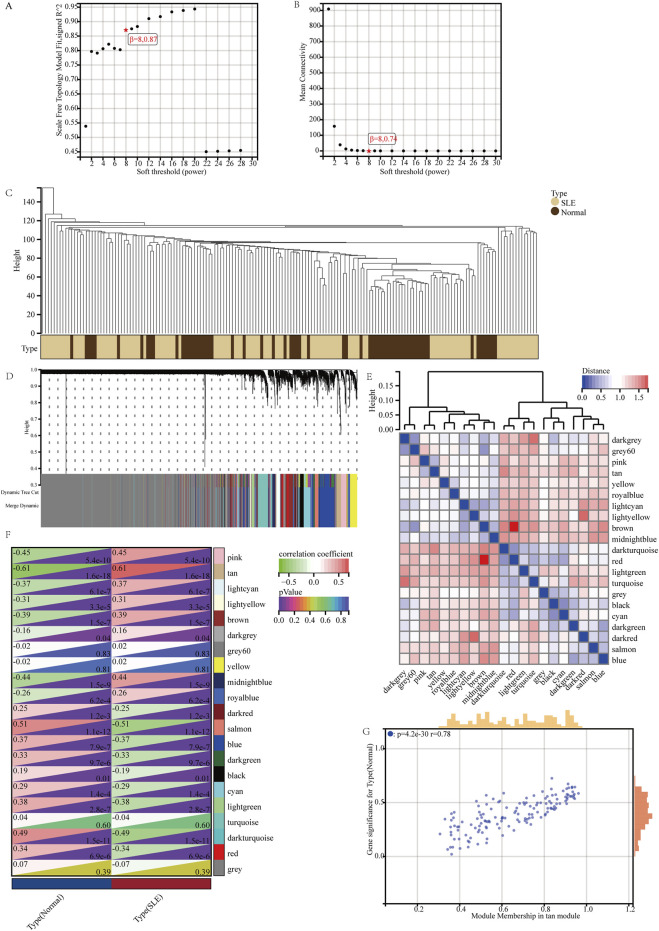
Weighted gene co-expression network analysis and key module identification. **(A, B)** β = 8 was selected as the soft threshold with the combined analysis of scale independence and average connectivity. **(C)** Clustering dendrogram of the SLE and control samples. **(D)** Gene co-expression modules represented by different colors under the gene tree. **(E)** Heatmap of eigengene adjacency. **(F)** Heatmap of the association between modules and SLE. The tan module was shown to be correlated significantly with SLE. Numbers at the top and bottom brackets represent the correlation coefficient and p-value, respectively. **(G)** Correlation plot between module membership and gene significance of genes included in the tan module.

WGCNA was applied to identify the most correlated gene modules in SLE. Based on scale independence and mean connectivity, β = 8.087 was selected as the soft threshold (Figures 3A, B). The cluster tree diagram of SLE and control was shown in Figure 3C. On this basis, 21 different color gene co-expression modules (GCMs, Figure 3D) were symbiosis. The correlation between SLE and the GCMs was shown in Figure 3F. The tan module (142 genes) had the highest correlation with SLE (r = 0.61, p = 1.6 × 10^−18^), which was the key gene module for subsequent analysis. We calculated the correlation between the tan module and gene significance, and there was a significant positive correlation between the two (r = 0.78, p = 4.2 × 10^−30^) (Figure 3G). Therefore, the tan module gene has the most significant correlation with SLE. The tan module has 142 genes.

### 3.4 Identification of candidate hub genes via machine learning

**FIGURE 4:**
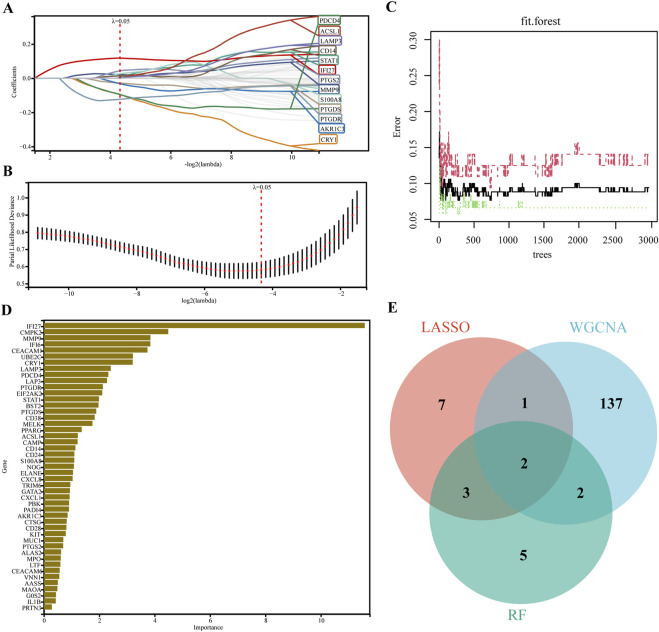
Machine learning in screening candidate diagnostic biomarkers. **(A, B)** Biomarkers screening in the Lasso model. The number of genes (n = 13) corresponding to the lowest point of the curve was the most suitable for SLE diagnosis. **(C)** The correlation plot between the number of RF trees and model error. **(D)** Genes ranked based on the importance score. **(E)** Venn diagram showed that two candidate diagnostic genes were identified via the above two algorithms.

LASSO regression and RF machine learning algorithms were used to identify potential biomarkers associated with SLE diagnosis. LASSO regression analysis of 46 genes after PPI analysis identified 13 genes that were closely related to the disease (Figures 4A, B). In RF algorithm, we assessed the importance of genes and screened 12 genes with importance greater than 2 (Figures 4C, D). The intersection of the 12 most important genes in RF, 13 genes in LASSO, and 142 genes in the key gene module of WGCNA identified two key genes (IFI27 and LAMP3) as key diagnostic markers for final validation (Figure 4E).

### 3.5 Diagnostic value assessment of hub genes and validation of hub genes in other datasets

**FIGURE 5:**
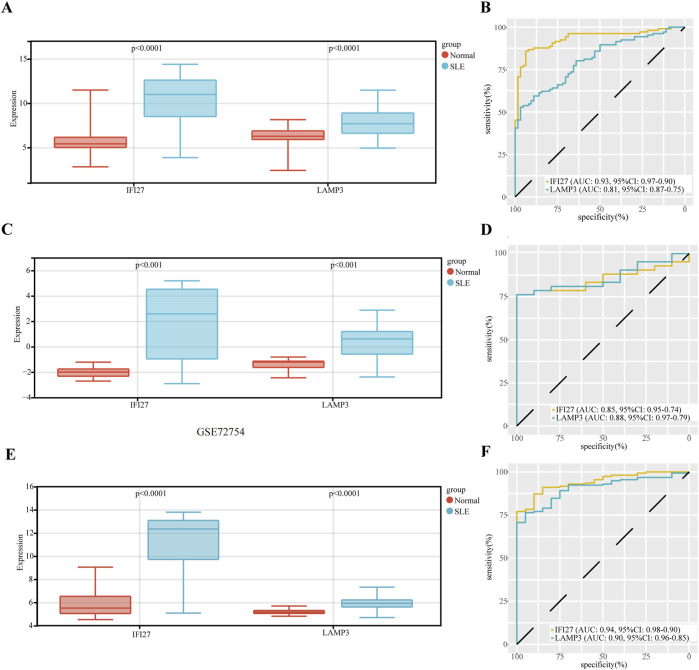
The diagnostic value evaluation of the two candidate diagnostic genes, and validation of hub genes in other datasets. **(A)** The expression of hub genes. **(B)** The ROC curve of each candidate gene. **(C)** Validation of the expression of hub genes in GSE72326 dataset. **(D)** The ROC curve of each candidate gene in GSE72326 dataset. **(E)** Validation of the expression of hub genes in GSE72754 dataset. **(F)** The ROC curve of each candidate gene in GSE72754 dataset.

The expression of hub genes (IFI27 and LAMP3) was upregulated in SLE (Figure 5A). The diagnostic specificity and sensitivity of each gene were evaluated by establishing an ROC curve, and the AUC and its 95% CI were calculated for each gene. The results were as follows (Figure 5B): IF27 (AUC 0.93, CI 0.97–0.90), LAMP3 (AUC 0.81, CI 0.87–0.75). All candidate biomarkers had high diagnostic value for SLE. The expression of key genes IIF27 and LAMP3 in SLE were further verified in GSE72326 dataset (177 cases, including SLE 157 cases, 20 normal controls) and GSE72754 dataset (52 cases, including SLE 42 cases, 10 normal controls), respectively. The expression trend was consistent in both validation sets (Figures 5C, E). For GSE72326 (Figure 5D), IF27 (AUC 0.94, CI 0.98–0.90) and LAMP3 (AUC 0.90, CI 0.96–0.85). For GSE72754 dataset (Figure 5F), IF27 (AUC 0.85, CI 0.95–0.74), LAMP3 (AUC 0.85, CI 0.95–0.74). All candidate biomarkers have high diagnostic value for SLE.

### 3.6 Immune cell infiltration analysis

**FIGURE 6:**
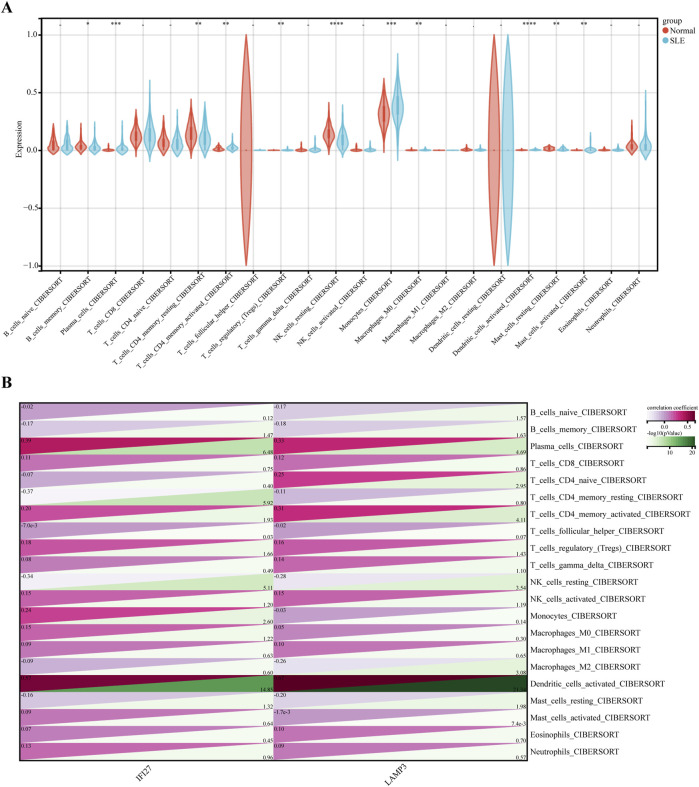
Immune cell infiltration analysis between SLE and control. **(A)** Comparison regarding the proportion of 22 kinds of immune cells between SLE and control groups visualized by the vioplot. **(B)** Correlation analysis between 22 immune cell types and Hub genes in SLE.

Functional enrichment analysis indicated that the immune system plays a crucial role in the development of SLE, and therefore, immune infiltration analysis can better explore the role of immunity in SLE. The gene expression levels of the SLE dataset were analyzed using the CIBERSORT algorithm to investigate the differences in immune infiltration of 22 types of immune cells, and it was found that 11 types of immune cells showed expression differences between the SLE and normal group. Box plots showed that compared with the normal group, the levels of Plasma cells, activated CD4 memory T cells, regulatory T cells (Tregs), Monocytes, M0 Macrophages, activated Dendritic cells, and Activated Mast cells were higher in the SLE group, while the levels of Memory B cells, Resting CD4 memory T cells, Resting NK cells, and Resting Mast cells were lower (Figure 6A).

Further exploration of the correlation between biomarkers and differential immune cells revealed that IFI27 is positively correlated with the level of Activated Dendritic cells, Monocytes, Tregs, Activated CD4 memory T cells, and Plasma cells, while it was negatively correlated with the level of Resting Mast cells, Resting NK cells, Resting CD4 memory T cells, and Memory B cells. LAMP3 was positively correlated with the Activated Dendritic cells, Tregs, Activated CD4 memory T cells, Naïve CD4 T cells, and Plasma cells, while it was negatively correlated with the Resting Mast cells, M2 Macrophages, Resting NK cells, Memory B cells, and Naïve B cells (Figure 6B).

### 3.7 Construction of cirRNA-miRNA-mRNA network

**FIGURE 7:**
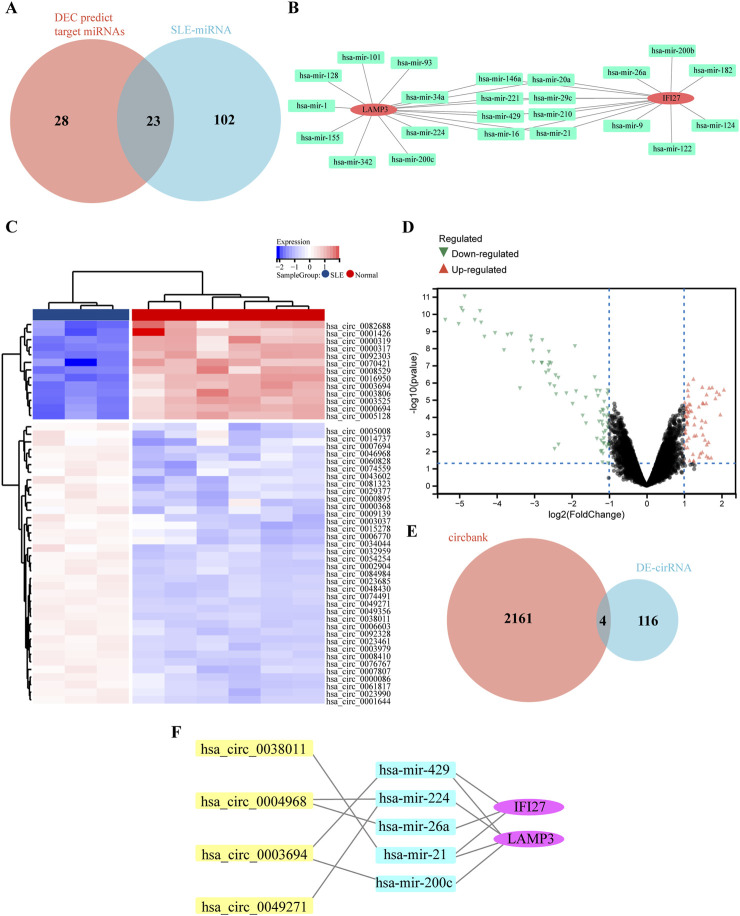
Construction of circRNA-miRNA-mRNA network. **(A)** The overlap of DECs predicted target miRNAs and SLE related miRNAs via Wenn diagram. **(B)** The 23 common miRNAs identified from the intersection of genes in DEG predicted target miRNAs and SLE related miRNAs. **(C)** The heat map of DECs in GSE84655 dataset. **(D)** The volcano map of DECs in GSE84655 dataset. Upregulated genes were marked in red; downregulated genes were marked in green. **(E)** The overlap of predicted DECs in the circbank database and DECs in GSE84655 dataset via Wenn diagram. **(F)** The circRNA-miRNA-mRNA network.

Non-coding RNA (ncRNA) plays an important role in the pathogenesis of SLE, including long non-coding RNA (lncRNA), miRNA and circRNA. Using the miRNet database, 51 miRNAs related to IFI27 and LAMP3 were predicted, and 125 miRNAs related to SLE were selected from the HMDD v4.0 database. The intersection of the two yielded 23 miRNAs (Figures 7A, B). Through the circbank database, 2,165 circRNAs were predicted. R software was used to analyze the GSE84655 dataset, and differentially expressed circRNAs (DECs) were identified with a P-value <0.05 and a |log2 Fold change (FC)|≥1, resulting in a total of 120 DECs (Figures 7C, D). After the intersection, 4 circRNAs were obtained (Figure 7E). The final constructed ceRNA network consists of 4 circRNAs, 5 miRNAs, and 2 mRNAs (Figure 7F).

### 3.8 The expression of key genes verification in clinical samples

**FIGURE 8:**
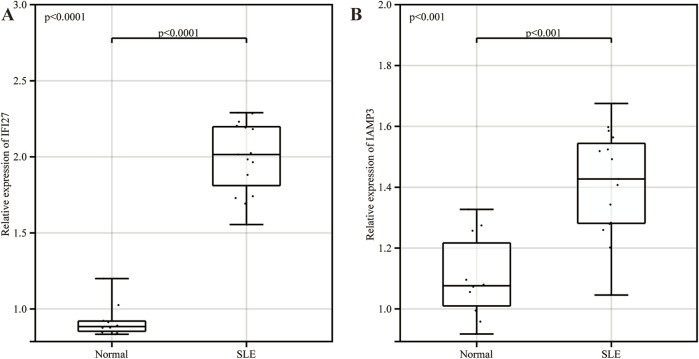
The expression of key genes verification in clinical samples. **(A)** The expression of IFI27 in clinical samples. **(B)** The expression of LAMP3 in clinical samples.

The results of real-time quantitative PCR showed that IFI27 and LAMP3 were highly expressed in SLE group compared with normal group, and the differences were statistically significant (Figure 8).

### 3.9 GSEA analysis of biomarkers

**FIGURE 9:**
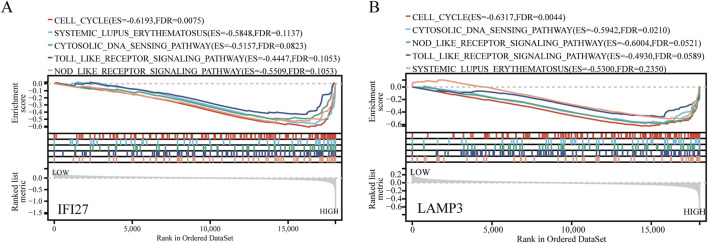
GSEA analysis for IFI27 and LAMP3. **(A)** GSEA analysis for IFI27. **(B)** GSEA analysis for LAMP3.

To further explore the possible mechanisms associated with the newly identified biomarkers, SLE samples were divided into high expression and low expression groups according to the median value of marker gene expression, and GSEA enrichment analysis was performed. The results showed that IFI27 and LAMP3 were mainly enriched in cell cycle, Systemic Lupus Erythematosus, cytosolic DNA sensing pathway, toll-like receptor (TLR) signaling pathway and nod-like receptor (NLR) signaling pathway (Figure 9).

## 4 Discussion

Systemic Lupus Erythematosus is an autoimmune disorder where the immune system abnormally attacks the body’s cells and tissues. It can manifest acutely or subtly, leading to damage across various tissues and organs (Siegel and Sammaritano, 2024). Currently, antinuclear antibodies, anti-double-stranded DNA antibodies, and complement activation are frequently utilized for diagnosing or assessing SLE disease activity (Anis et al., 2023). However, due to the disease’s complexity, using a single biomarker for SLE assessment is quite difficult. Thus, there is an urgent need for deeper research into diagnostic biomarkers for SLE.

Mitochondria, maternally inherited organelles, participate in numerous biological processes such as cellular energy metabolism and programmed cell death (Poltorak, 2022). Autoantibodies against mitochondria and its components, such as anti-cardiolipin antibodies, anti-mitochondrial antibodies, anti-mitochondrial RNA antibodies, anti-mitochondrial outer membrane, and anti-mitochondrial DNA antibodies, have been identified in SLE patients’ serum (Chen and Tsokos, 2022; Gomez-Banuelos et al., 2023). To some extent, their serum levels correlate with the degree of tissue damage and SLE activity indicators like the SLEDAI score (Becker et al., 2019). There is a close relationship between mitochondria and programmed cell death, mitochondria play a pivotal role in apoptosis, necrosis, and other forms of cell death (Flores-Romero et al., 2023). They also interact with other organelles, such as the endoplasmic reticulum and the nucleus, to regulate apoptosis. Mitochondrial damage and dysfunction can lead to insufficient ATP supply and excessive ROS accumulation, promoting necrosis. During pyroptosis, mitochondrial membrane rupture and the release of inflammatory factors are critical steps; while in ferroptosis, mitochondria influence cell fate by modulating iron metabolism and ROS production (Vringer and Tait, 2023; Flores-Romero et al., 2023; Bock and Tait, 2020).

Programmed cell death (PCD) critically contributes to SLE pathogenesis. Abnormal apoptosis and defective clearance of apoptotic debris lead to autoantigen exposure, immune activation, autoantibody production, immune complex formation, and tissue damage (Xu Y. et al., 2022; Sun et al., 2021). Neutrophils in SLE release nuclear and mitochondrial DNA (mtDNA), linked to Gasdermin D (GSDMD)-mediated pyroptosis; oxidized mtDNA (Ox-mtDNA) further enhances GSDMD-N oligomerization, amplifying pyroptotic cell death (Miao et al., 2023; Xin et al., 2024). Ferroptosis, driven by elevated autoantibodies and type I interferons (IFN) in SLE serum, is implicated in neutrophil death and disease progression (Ohl et al., 2021; Zhao G. et al., 2024). Different types of cells exhibit different sensitivities and characteristics to a certain type of cell death, and there are complex interconnections between different PCDs within different cells, accelerating cell death and promoting the progression of SLE (Xu Y. et al., 2022). Further research on the interconnections between various forms of cell death and mitochondrial function in SLE will help to reveal the pathogenic mechanisms and new therapeutic targets.

Our study involved analyzing DEGs from three SLE GEO databases and intersecting them with 1,548 programmed cell death-related genes and 1,136 mitochondrial-related genes identified from the MitoCarta 3.0 database, resulting in the identification of 47 DEGs related to PCD and 14 DEGs related to mitochondria. Through bioinformatics and machine learning, we discovered that the common genes IFI27 and LAMP3, which are related to PCD and mitochondria, play an important role in SLE. The diagnostic efficacy of IFI27 and LAMP3 was evaluated using ROC in a training set and two validation sets, indicating that both IFI27 and LAMP3 have good diagnostic efficacy for SLE.

IFI27 (Interferon Alpha Inducible Protein 27) is associated with various biological functions such as apoptosis, autophagy, pyrolysis, and immune regulation, IFI27 is highly expressed in brown adipose tissue and is closely related to mitochondrial function and the thermogenic capacity of adipocytes (Cui et al., 2023). The role of IFI27 in SLE is mainly related to its function in the interferon (IFN) signaling pathway. IFI27 is one of the interferon-stimulated genes, and its expression level is usually elevated in SLE patients and is related to disease activity (Cecchi et al., 2024). Studies have shown that high expression of IFI27 is related to synovitis in SLE patients (Nzeusseu Toukap et al., 2007). In addition, different B cell subsets in SLE patients have different response characteristics to type I and type III IFNs, and patients with high levels of IFI27 have significantly higher levels of IFN-α in peripheral blood mononuclear cells (PBMCs) than patients with low levels of IFI27 or healthy donors (Song et al., 2023).

LAMP3 (Lysosome-Associated Membrane Protein 3) is a glycosylated membrane protein typically expressed in lymphoid organs and serves as a marker for mature human dendritic cells (DCs), with its expression being upregulated during DC activation and maturation (Malaguarnera et al., 2017). The increase in LAMP3 expression is associated with the development of Sjögren’s Syndrome (SS) (Tanaka et al., 2020). Research indicates that overexpression of LAMP3 can lead to the relocalization of lysosomal cathepsins to the cytoplasm by increasing the permeability of the lysosomal membrane, which may trigger instability in the autophagic flux and activation of caspases, leading to apoptosis (Nakamura et al., 2023). Additionally, LAMP3 may be involved in regulating the fusion of autophagosomes with lysosomes, thereby affecting the degradation and renewal of mitochondria (Tanaka et al., 2023). The role of LAMP3 in PCD and mitochondrial function is multifaceted, involving various aspects such as apoptosis, autophagy, and tumor immunity (Tanaka et al., 2022).

Immune infiltration analysis found that there were differences in the expression of 11 types of immune cells between SLE and normal groups. Correlation analysis revealed that IFI27 is positively correlated with 5 immune cells, while negatively correlated with 4 immune cells. LAMP3 is positively correlated with 5 immune cells, and negatively correlated with 5 immune cells. In SLE pathogenesis, plasmacytoid dendritic cells (pDCs) critically contribute through TLR7/9-mediated recognition of immune complexes, triggering excessive type I interferon (particularly IFN-α) production via the JAK-STAT pathway. SLE patients exhibit reduced peripheral blood pDCs, with significant accumulation in affected tissues (e.g., kidneys, skin) (Soni et al., 2020). Monocytes demonstrate pathological polarization in SLE: classical monocytes exhibit pro-inflammatory properties supporting macrophage differentiation, while non-classical monocytes display Th17-regulatory phenotypes. Both subsets facilitate autoantibody production through B-cell interaction and immune complex formation (Hirose et al., 2019; Merino-Vico et al., 2023). Treg cell dysfunction in SLE manifests through altered expression of co-stimulatory molecules (CTLA4, PD-1) and cytokines (IL-2, IL-10, TGF-β), impairing their immunosuppressive capacity (Bertsias et al., 2009; Tenbrock and Rauen, 2022). Disease activity correlates with CD4^+^CXCR5^−^PD1^+^ memory T cell expansion (Sagrero-Fabela et al., 2023). Pathogenic plasma cells sustain autoantibody production, while mast cells activate through IgE/FcεRI-mediated pathways (Wang et al., 2022). SLE patients show reduced circulating NK cells and complex M2 macrophage involvement in disease progression (Zhao L. et al., 2024).

The cirRNA-miRNA-mRNA network constitutes the mechanism of the competing endogenous RNA (ceRNA) network (Wu et al., 2024; Zhou et al., 2023; Kohansal et al., 2024). In SLE, some circRNAs may participate in the regulation of immune responses and inflammatory processes by adsorbing miRNAs, changing the availability of miRNAs, and thereby affecting the expression of related genes. hsa_circ_0045272 can act as a sponge for miR-6127, regulating T cell apoptosis and IL-2 secretion (Li et al., 2018). In SLE, circRNAs participate in the occurrence and development of the disease through various mechanisms, with upregulated expression of circADCY9 and circGARS and downregulated expression of circMCTP2. The expression levels of these circRNAs are correlated with SLE disease activity Index (SLEDAI) scores and complement C3 levels, suggesting that they may be involved in regulating disease activity in SLE (Zhao et al., 2022). miRNAs are involved in the regulation of immune cells in SLE, including T cells, B cells, and dendritic cells, affecting the pathogenesis of SLE by influencing the function and interaction of these cells (Chi et al., 2021). miR-146a is abnormally expressed in SLE patients and may affect inflammatory responses and cytokine production, thereby affecting disease activity. miRNAs participate in the cell survival and death processes in SLE by regulating the expression of genes related to apoptosis (Hsieh et al., 2022). miRNAs can also affect various signaling pathways involved in SLE, such as the JAK-STAT pathway and the NF-κB pathway, which play key roles in the activation of immune cells and inflammatory processes (Wang et al., 2021). Studies have shown that the levels of miR-200c and miR-429 in the serum and urine of SLE patients were lower than those of control group and the expression of miR-21 and miR-224 was upregulated and the expression of miR-26a was downregulated in kidney tissue of SLE patients (Wang et al., 2011). In our study, we found 5 miRNAs related to IFI27 and LAMP3, which were hsa-mir-429, hsa-mir-224, hsa-mir-26a, hsa-mir-21, and hsa-mir-200c. Four circRNAs were obtained by the intersection of miR-predicted circRNAs and DECs, namely, hsa_circ_0038011, hsa_circ_0049271, hsa_circ_0004968, and hsa_circ_0003694. Thus, a ceRNA regulatory network consisting of 2 mRNAs, 5 miRNAs and 4 circRNAs was formed.

GSEA indicates that in SLE patients, abnormalities in the cell cycle may lead to abnormal activation, proliferation, and apoptosis of immune cells, thereby affecting the disease progression. Variations in the cell cycle-related gene NCF1 have been found to be associated with autoimmune characteristics of SLE (Olsson et al., 2017). Cyclic GMP-AMP synthase (cGAS), as the main cytoplasmic DNA sensor, can recognize double-stranded DNA (dsDNA) in the cytoplasm. Upon activation, it produces the second messenger cGAMP, which subsequently activates stimulator of interferon genes (STING). In SLE patients, self-DNA released during cell death may be recognized by cGAS, activating the cGAS-STING pathway, triggering the production of type I interferons and inflammatory cytokines, which is related to the autoimmune response in SLE. In addition, the abnormal activation of the cGAS-STING pathway is also related to B cell differentiation and T cell activation in SLE, which play a key role in the adaptive immune response (Feng et al., 2024). The activation of the Toll-like receptor signaling pathway can lead to the production of various cytokines and chemokines, which play a role in the inflammatory response and tissue damage of SLE. Therefore, the regulation of the Toll-like receptor signaling pathway has become a potential target for the treatment of SLE (Fillatreau et al., 2021). The NOD-like receptor signaling pathway, especially the NLRP3 inflammasome, is associated with the occurrence and development of the disease and is related to the degree of kidney damage in SLE patients (Oliveira et al., 2021). It plays an important role in the pathogenesis of SLE and is a potential target for future therapeutic strategies.

Although this study has been validated through clinical samples, the limited sample size and lack of investigation into different disease stages of SLE restrict its comprehensiveness. Further research utilizing cell experiments and animal studies is required to elucidate the specific molecular mechanisms involved. Future studies may focus on exploring the roles of these molecules in SLE, aiming to provide novel strategies and therapeutic targets for the precise diagnosis and treatment of SLE.

In summary, this study identified two hub genes related to programmed cell death and mitochondrial function, namely, IFI27 and LAMP3, which may be regulated by a ceRNA network composed of 4 circRNAs and 5 miRNAs and participate in the pathogenesis of SLE. A variety of immune cells were also related to these two biomarkers. This study provides new insights and potential therapeutic targets for elucidating the role of programmed cell death and mitochondrial function in SLE, and the two biomarkers can lay a theoretical foundation for the diagnosis and treatment of SLE.

## Data Availability

The datasets presented in this study can be found in online repositories. The names of the repository/repositories and accession number(s) can be found in the article/Supplementary Material.
