# Supplementary figures and images for: Identification of mitochondrial function and programmed cell death associated key biomarkers and the circRNA-miRNA-mRNA regulatory network in systemic lupus erythematosus
Source: Front Mol Biosci. 2025 Apr 14;12:1586294. doi: 10.3389/fmolb.2025.1586294 (PMC12034568; doi:10.3389/fmolb.2025.1586294)

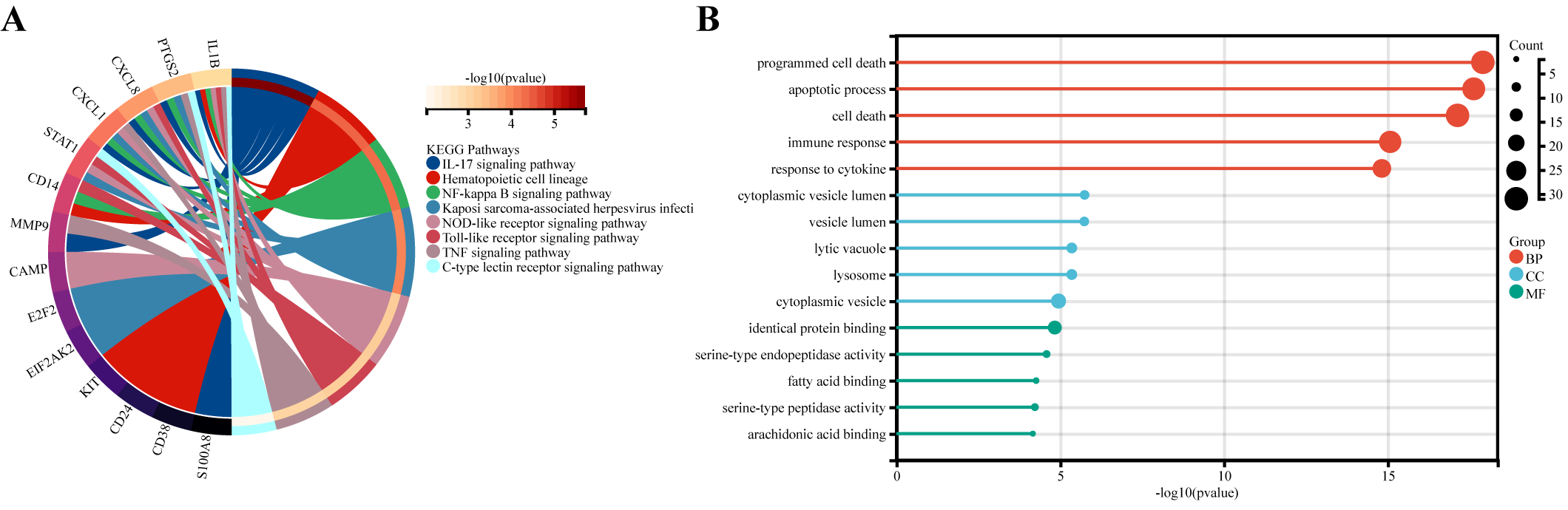

Supplement: Supplementary file 1 [file Image3.tif]

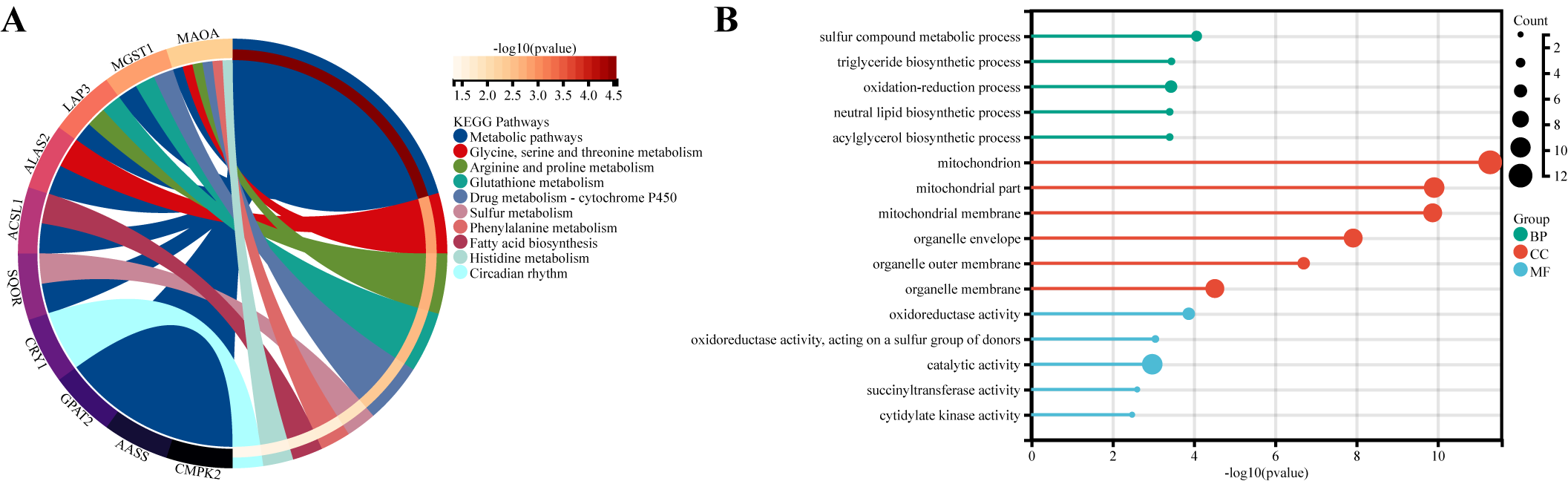

Supplement: Supplementary file 2 [file Image2.tif]

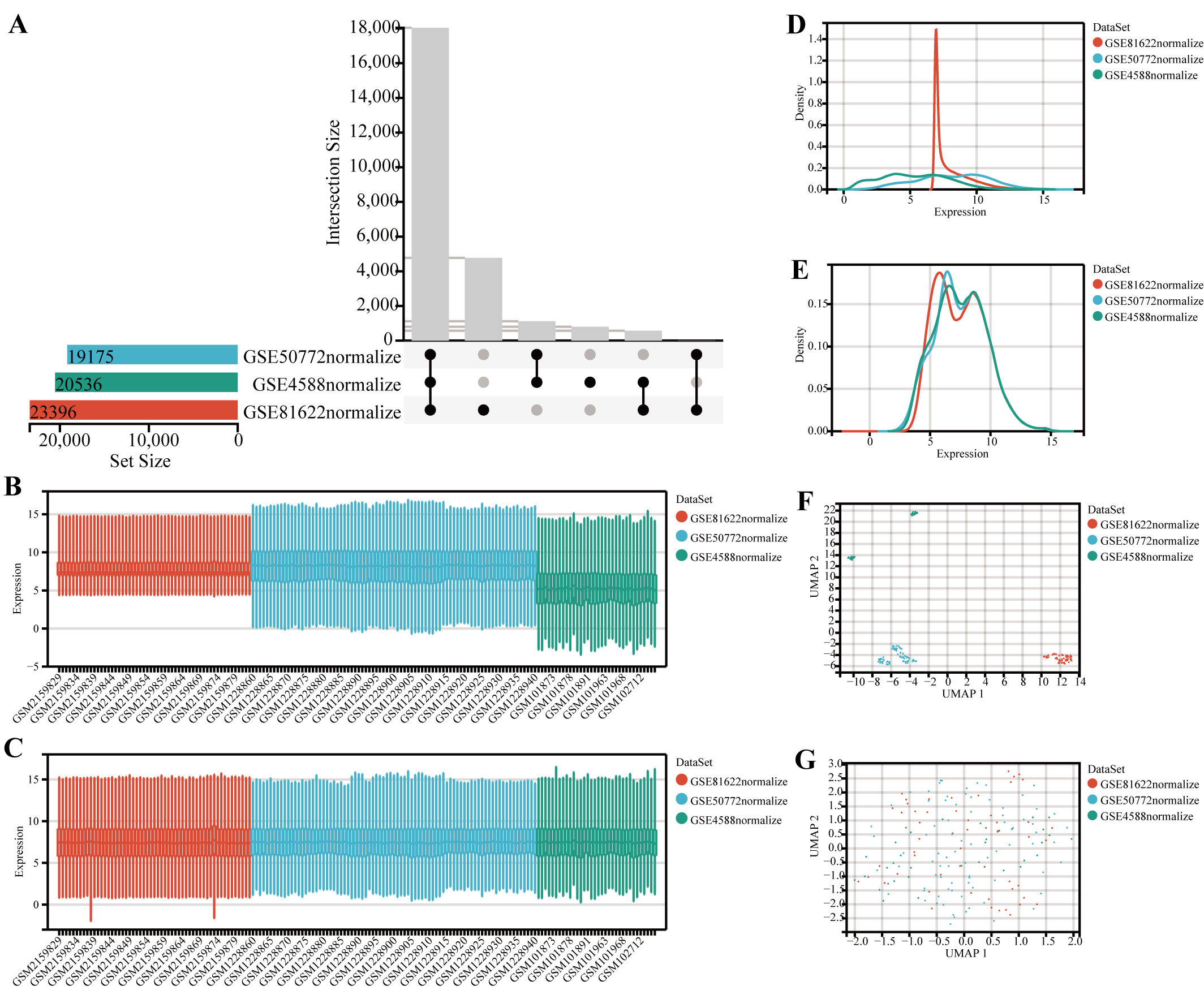

Supplement: Supplementary file 3 [file Image1.tif]
